# Alkahest NuclearBLAST : a user-friendly BLAST management and analysis system

**DOI:** 10.1186/1471-2105-6-147

**Published:** 2005-06-15

**Authors:** Stephen E Diener, Thomas D Houfek, Sam E Kalat, DE Windham, Mark Burke, Charles Opperman, Ralph A Dean

**Affiliations:** 1 Fungal Genomics Laboratory, Center for Integrated Fungal Research, North Carolina State University, Raleigh, NC 27695, USA; 2 Plant Nematode Genetics Group, North Carolina State University, Raleigh, NC 27695, USA

## Abstract

**Background -:**

Sequencing of EST and BAC end datasets is no longer limited to large research groups. Drops in per-base pricing have made high throughput sequencing accessible to individual investigators. However, there are few options available which provide a free and user-friendly solution to the BLAST result storage and data mining needs of biologists.

**Results -:**

Here we describe NuclearBLAST, a batch BLAST analysis, storage and management system designed for the biologist. It is a wrapper for NCBI BLAST which provides a user-friendly web interface which includes a request wizard and the ability to view and mine the results. All BLAST results are stored in a MySQL database which allows for more advanced data-mining through supplied command-line utilities or direct database access. NuclearBLAST can be installed on a single machine or clustered amongst a number of machines to improve analysis throughput. NuclearBLAST provides a platform which eases data-mining of multiple BLAST results. With the supplied scripts, the program can export data into a spreadsheet-friendly format, automatically assign Gene Ontology terms to sequences and provide bi-directional best hits between two datasets. Users with SQL experience can use the database to ask even more complex questions and extract any subset of data they require.

**Conclusion -:**

This tool provides a user-friendly interface for requesting, viewing and mining of BLAST results which makes the management and data-mining of large sets of BLAST analyses tractable to biologists.

## Background

Recently the number of research groups generating sequence data such as expressed sequence tags (EST) and bacterial artificial chromosome (BAC) ends has increased dramatically due to dropping costs of obtaining DNA sequence. The size of groups involved in such projects ranges from members of sequencing centers with a large bioinformatic support staff to individual researchers with little data management expertise. As it is now typical for even the smallest projects to generate hundreds to thousands of sequences, tools that streamline annotation, data mining and data management have become increasingly important to biologists without large bioinformatic support staffs.

Annotation of a large set of sequences typically involves attempting to subdivide the sequences into common functional categories. The usual course of action followed is to perform one or more BLAST searches against databases to reveal homologies with sequences previously functionally annotated. Using these homologies, categorical annotations such as Gene Ontology terms can be associated with the novel sequences and used to evaluate the sequence dataset. When dealing with the large numbers of sequence reads generated by these projects, storage and compilation of flatfile BLAST results related to this endeavor becomes rather cumbersome. Furthermore, comparative analysis of gene complement between related organisms can provide a number of insights into many areas of biology. Identification of a subset of genes which exist in a pathogen but not in a related non-pathogenic organism can provide targets for other functional analyses such as gene disruption experiments. While there exist other BLAST clustering packages, such as BeoBLAST or provided by NCBI, they do not provide a free open-source complete result management package with storage of results in a relational database (RDMS) [1,7]. The ability to easily locate, data-mine and perform comparative analysis on BLAST results can be substantially simplified through the use of a RDMS. In order to facilitate sequence analysis and comparative genomics we have created NuclearBLAST.

## Implementation

The primary design goals for NuclearBLAST were to provide biologists a centralized system where BLAST results can be easily created and retrieved, a relational database storage system which can be easily mined for comparative analyses, and a program which would take advantage of clustered computing resources to increase the throughput of large BLAST jobs. A secondary objective was to design a solution which was open-source and freely available. This led us to preferentially utilizing a number of open-source software packages in the implementation of NuclearBLAST including BioPerl, Apache, PHP and MySQL as well as using Linux as the base operating system [2-5].

A web browser was chosen as the interface for NuclearBLAST since it provides a widely used, recognizable, globally available, platform independent interface. The Apache web server provides access control and encrypted connections if the user desires to secure their installation. BioPerl provides a parsing module for the returned BLAST results which are then loaded into the MySQL database. All information about requested BLAST searches are loaded into the database and the status of each query in a job is tracked in the database. This client server design of the program allows for the use of multiple computers performing the BLAST analyses in a clustered environment by using a job management software package such as PBS (Portable Batch System) [6]. NuclearBLAST can be clustered with as little as several lab computers used as worker nodes in their down time or as much as a dedicated compute farm. A minimal installation of NuclearBLAST requires a typical workstation machine acting as both the server and the worker.

In order to keep the MySQL database to a manageable size and reduce redundant data in the system we opted to use BLAST's database format as the only store of sequence information in the program. Only the sequence names within each dataset and a minimal amount of metadata are stored in the MySQL database. We also minimized the database size by storing all result statistics in the database but excluding the actual alignments. When requested, the alignments are recreated on the fly by extracting the two sequences from the BLAST databases and blasting them against each other using bl2seq.

## Results

NuclearBLAST is a free open source batch BLAST analysis, storage and management system which provides a simple platform for performing BLAST analyses and mining of the results. NuclearBLAST is written in Perl which drives NCBI's *blastall *application to conduct its analyses and store all BLAST results in a MySQL database [7]. Users may import datasets, request analyses, and view results in the PHP web interface through their browser. Command-line programs support more complex or special-purpose queries. NuclearBLAST may be installed on a single machine, or it may be set up to distribute BLAST analyses to a cluster of machines by way of a batch queuing system.

Sequence files are imported in FASTA format. NuclearBLAST uses NCBI's *formatdb *utility to parlay sequences into the format required for *blastall *targets. When importing a sequence data set into NuclearBLAST, a user specifies whether the sequence may be used in subsequent searches as a query, a target, or both. Specifying 'both' allows easy reciprocal BLASTs between smaller datasets. These parameters can be set to allow users access to large datasets as targets but not as queries. This guards against the casual mistake of querying very large datasets, such as the Genbank non-redundant protein database, against much smaller datasets (rather than vice versa). If unnoticed, such a mishap can divert a laboratory's analysis system for extended periods of time.

**Figure 1:**
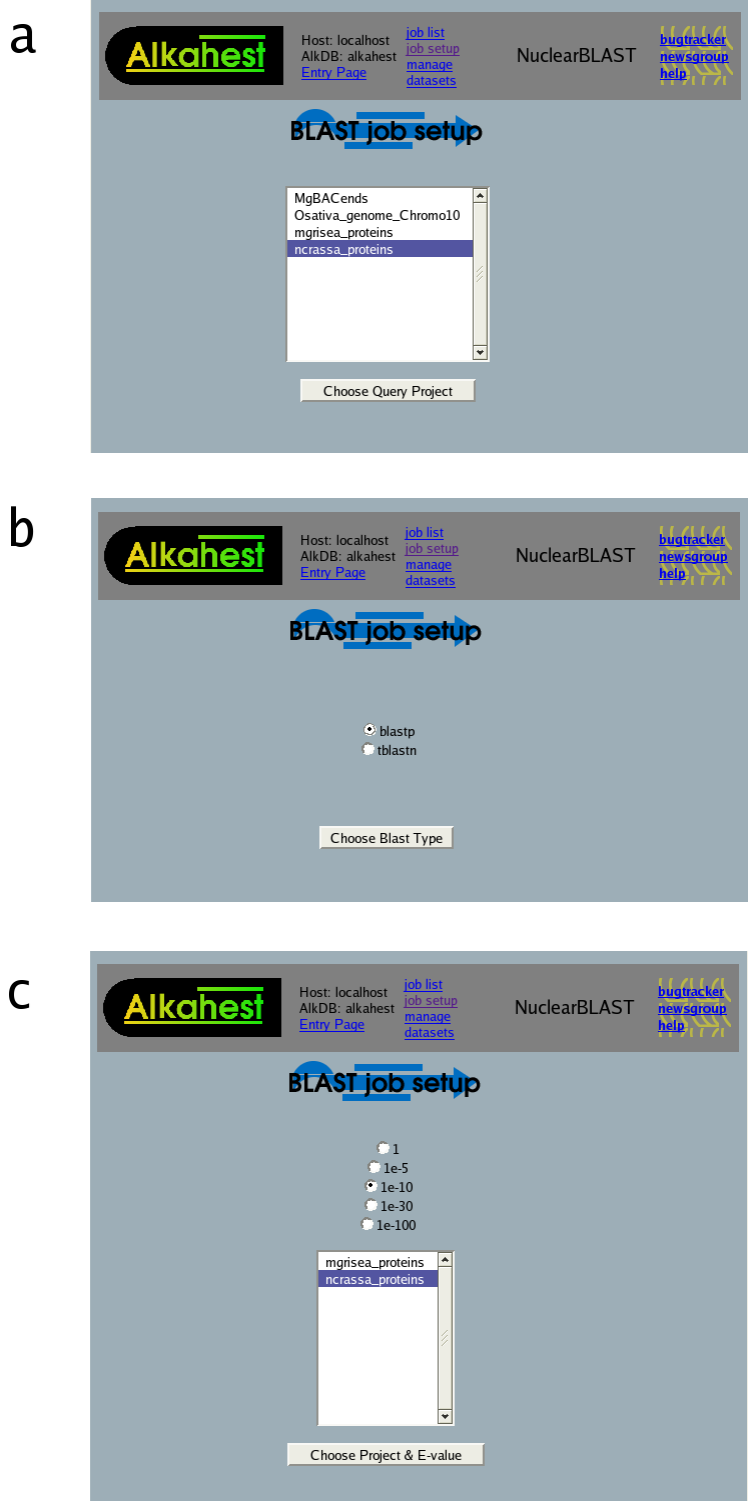
Screenshots of the NuclearBLAST Job Request Wizard. Panel **a **shows the set of possible query sets in the system. Upon choosing one of these, the appropriate BLAST sub-programs are made available in Panel **b**. After choosing one of the sub-programs, Panel **c **arises which allows you to limit the e-value of stored results and gives choices of BLAST target databases in the system which are acceptable based upon prior decisions.

Requesting batch BLAST jobs through the web interface is facilitated by a job request wizard which guides the user through the process. The user first chooses a query dataset (from datasets designated as allowable queries) (Fig 1a). The choice of query (specifically, whether it is a nucleotide or protein sequence) automatically limits the menu of programs of the BLAST "family" (BLASTN, TBLASTX, etc) to those appropriate for the type of query sequence (Fig 1b). The user's choice of program dictates the sequence type (nucleotide/protein) appropriate for targets; in the next stage of the wizard, when the user selects a target sequence dataset, only choices of the appropriate type appear on the menu (Fig 1c). The job request is completed by specifying an e-value threshold for returned results. (More advanced BLAST parameters can be stipulated by using NuclearBLAST's command-line request utility).

**Figure 2:**
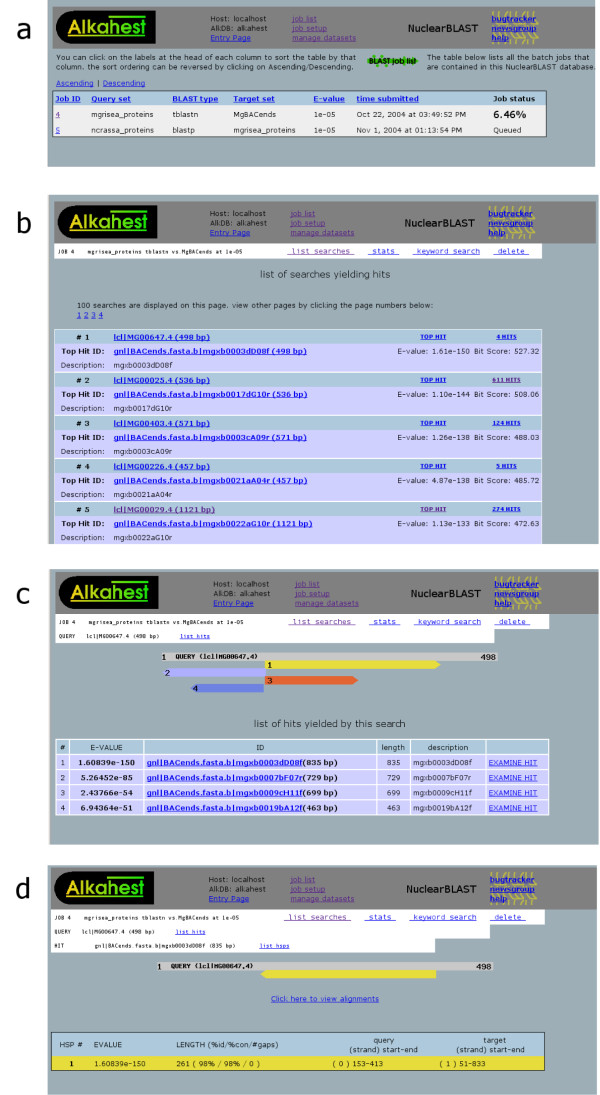
Screenshots of the NuclearBLAST web interface. Panel **a **shows the requested jobs and their progress. Clicking on a hyperlinked job number brings up a page, panel **b**, containing the results of multiple query sequences within that requested job. The link on this page showing the number of hits fetches the location and statistics of multiple hits to that query sequence, panel **c**. Examining a hit further provides a view of the HSP locations and statistics for a single hit, panel **d**. Hyperlink of a sequence name retrieves a page with information about that particular sequence.

**Figure 3:**
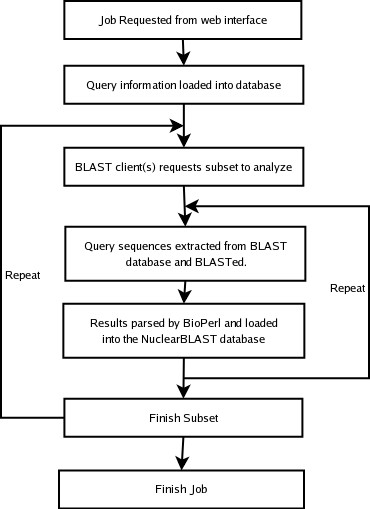
Flow of a NuclearBLAST Job. Illustration of the program's work flow when progressing through a requested BLAST analysis

After a request is submitted, the status of the job can be continually monitored on the job's main page (Fig 2a). Invocations of *blastall *are scheduled by a simple first-in-first-out scheme. NuclearBLAST parses *blastall's *output files using BioPerl and loads their constituent data into the database [8]. The work flow of a job from request to completion can be seen in Figure 3.

Results of a completed batch BLAST job can be browsed as ordered by e-value in a paginated fashion with the top hit available on the main result page (Fig 2b). Clicking on the query calls up a page which shows all hits to that sequence found, along with a graphical view of where each hit aligned to the sequence (Fig 2c). Each hit further contains a link to a page showing the alignment of the HSPs and other associated statistic (Fig 2d). In addition to ordered hierarchical browsing, NuclearBLAST offers a facility for searching for strings within text fields associated with the job (names or descriptions of queries or hits). This can help researchers quickly find certain selected targets amidst a large volume of search results.

In addition to providing an easy web-based method of perusing BLAST results, NuclearBLAST supplies a number of command line scripts allowing expanded access to search results in the database. One such script exports a job's results to a tab delimited file for inclusion in a spreadsheet or publication. Another determines reciprocal best hits between two datasets that have been reciprocally BLASTed. A third script may be used to transfer Gene Ontology (GO) annotations to query sequences that have matches against a database annotated with these terms. As Compugen has made available GO annotations for much of the Genbank non-redundant protein database at the Gene Ontology website, one can easily annotate a large set of sequences with GO terms using NuclearBLAST [9,10]. This provides the researcher an easy way to categorize their sequences into functional groupings. Future work on NuclearBLAST will extend the mining capabilities available on the command line as well as through the web interface and also expand integration with clustering software.

## Conclusion

NuclearBLAST provides a powerful tool to biologists for data mining and comparative genomic analysis of generated sequence. It has shown its utility in prior studies [11]. This program provides a simple interface for performing large batch BLAST searches, effectively manages a large number of search results, presents those results in an intelligibly browsable format, and provides an extensible platform for more thorough data mining of BLAST results.

## Availability and requirements

The program, source and full documentation for installation and use are available at  as well as in the additional file section [see Additional File 1]. Installation of the software is walked through in the documentation and requires personnel with Unix/Linux installation experience. The software is licensed under the GNU GPL and requires PHP, Perl, Apache, and MySQL.

## Supplementary Material

Additional File 1 The program, source and full documentation for installation are included. Click here for file
