# Supplementary material for: Alkahest NuclearBLAST : a user-friendly BLAST management and analysis system
Source: BMC Bioinformatics. 2005 Jun 15;6:147. doi: 10.1186/1471-2105-6-147 (PMC1181624; doi:10.1186/1471-2105-6-147)
Supplement: Additional File 1 — The program, source and full documentation for installation are included. [file 1471-2105-6-147-s1.gz › alkahest-0.7.5/www/help/index.html]

# Alkahest v0.7.4 Complete Documentation

| Manager's Guide | A quick introduction to the Alkahest system, written for decision-makers thinking of implementing Alkahest. This should help you to begin to decide whether Alkahest is right for you. |
| --- | --- |
| Installation Guide | A guide to installing and configuring Alkahest and its prerequisite software. |
| User's Guide | A guide for the everyday use of Alkahest. Explains WWW interface and command-line directives. |
| Administrator's Guide | A guidebook for the Alkahest system administrator. Covers security vulnerabilities, backup procedures, sequence data pipeline configuration, GenBank BLAST dataset updates, system troubleshooting, etc. |
| Developer's Guide | A guidebook for those intrepid (or damned!) souls who need to know what goes on under the covers. |

Acknowledgements
