# Supplementary material for: Alkahest NuclearBLAST : a user-friendly BLAST management and analysis system
Source: BMC Bioinformatics. 2005 Jun 15;6:147. doi: 10.1186/1471-2105-6-147 (PMC1181624; doi:10.1186/1471-2105-6-147)
Supplement: Additional File 1 — The program, source and full documentation for installation are included. [file 1471-2105-6-147-s1.gz › alkahest-0.7.5/www/help/managers_guide.html]

# Alkahest v0.7.4 Manager's Guide

Alkahest Overview

Alkahest Interceptor

Alkahest NuclearBLAST

Alkahest
Overview

It is imporant to be clear about what Alkahest is. Alkahest is
free open source systems software for high-volume DNA sequencing
projects. The cost of in-house systems development to support a
sequencing project can be very steep; expensive too are proprietary
software suites, and off-site services. What is needed are free and
open sequence data management and analysis platforms that research
groups can more or less easily configure or extend to meet their
needs. This version of Alkahest (version 0.7.4) is the fruit of
about two years of work by a handful of programmers collaborating
amongst several new high-throughput sequencing centers. All of our
centers are committed to sharing the products of their internal
development efforts with each other and with the community at large.
We, the programmers, founded The Alkahest Initiative
(www.alkahest.org) as the hub
for this collaboration.

We believe that we are well situated to extend and improve
Alkahest rapidly over the next several years. The core developers
have institutional support, so that much of the code they write at
work can flow immediately into the Alkahest development effort. More
programmers are becoming involved in the effort. For the first time
we think we can say with confidence, and not just with determination,
that Alkahest is here for the long term.

That's good, because we have a long way to go! “Release Early,
Release Often” is the watchword of our project. We want to get as
many people to try our system as possible, because their feedback
helps us to improve it. Our first major release, version 1.0.0, will
probably be available by 2004. We want version 1.0.0 to be
competitive with major commercial software systems for a broad array
of sequencing projects.

The Alkahest system is designed for the UNIX operating system. In
fact, our development so far has centered on the Linux operating
system, which itself is free. Most of the external software required
by Alkahest is free for everyone: all of it is free for academic
researchers.

An Alkahest system is installed upon one or more Linux computers,
but may be accessed by any number of computers across a TCP/IP
network (yea, even across the entire World Wide Web), because
Alkahest's primary interface is a “web application” which you
access through a web browser. Some administrative tasks must be
performed on the servers, but the web interface suffices for most
everyday tasks.

This version of Alkahest features two independent but
interoperable modules: “Alkahest Interceptor” and “Alkahest
NuclearBLAST”. You can use one or the other; or you can use them
both and jointly. It's time we introduced them.

Alkahest
Interceptor

Interceptor is Alkahest's high-throughput DNA sequence data
management module, probably suitable for most ABI3700 sequencing
projects. It has the name Interceptor because after it has been
configured to recognize a project's naming convention, it
automatically intercepts and processes volumes of trace files as soon
as they arrive. It calls upon PHRED and CROSSMATCH (freely available
to academic researchers but distributed separately) to base call and
screen sequences. Vector screening, contaminant screening, primer
and poly-A identification may be performed automatically. Trace
files and open text files are organized in the filesystem as they
arrive, with sequence and quality information deposited into a
relational database. Interceptor may be configured to send regular
digest summaries of its daily processing, and to fire off alert
emails in response to some suspicious quality control events or
trends. It allows you to oversee the progress of all of your
sequencing projects from anywhere using a web browser. (There are
some additional security pre.autions you need to take if you don't
want *just anyone* to be able
to admnister your Alkahest system remotely. Which you don't.)
The web interface brings together the quality statistics with
graphical annotations of artifactual sequence features, making it
easier for researchers to troubleshoot some common DNA preparation
problems. Alkahest effectively trims screened sequence and can
export project data in FASTA or .PHD format. If you are also
running Alkahest NuclearBLAST, you may take an Interceptor data set
and automagically import it into NuclearBLAST as a BLAST dataset.

Alkahest
NuclearBLAST

NuclearBLAST is Alkahest's batch BLAST homology search engine.
Using the web interface, users may create BLAST datasets from
uploaded FASTA files or from Interceptor project data, and may set up
batch BLAST searches between these datasets. NuclearBLAST then
dispatches these searches. With some elbow grease it can be attached
to a batch system for a computation farm, as the Fungal Genomics
Laboratory has done with PBS (Portable Batch System). Search results
are deposited in a relational database, and BLAST 'reports' are
viewed as dynamic web pages, with graphical diagrams highlighting
regions of homology. NuclearBLAST also offers simple keyword
searching and automatically generated statistical reports.

If either or both of these modules sound like they might be useful
to you, and if you can afford to be an early adopter (if you've got
someone with some computer expertise around who's willing to tinker
with as-yet-unperfected software), we encourage you to try us out.
Give the rest of this documentation (particularly the *Installation
Guide*, which tells you what
other hardware and software you'll need) a look. Feel free to
contact us through www.alkahest.org
if you have any questions or comments.
