# Supplementary material for: Alkahest NuclearBLAST : a user-friendly BLAST management and analysis system
Source: BMC Bioinformatics. 2005 Jun 15;6:147. doi: 10.1186/1471-2105-6-147 (PMC1181624; doi:10.1186/1471-2105-6-147)
Supplement: Additional File 1 — The program, source and full documentation for installation are included. [file 1471-2105-6-147-s1.gz › alkahest-0.7.5/www/help/installation_guide.html]

# Alkahest v0.7.4 Installation Guide

supported platforms

general recommendations

prerequisite software

Red Hat
v.8.0 installation walkthrough

Installing Bioperl

Configuring
NCBI BLAST

setting
environment variables

Setting
up a persistent system-wide shell environment variable

Setting
up the environmental variable for the Apache Web Server

Starting
Apache

Configuring cron

troubleshooting
installation problems

---

supported
platforms

Alkahest has been developed exclusively under Red Hat Linux,
beginning with version 7.0 and continuing up through version 8.0. As
of this writing, Red Hat v9.0 is being released. We have not tested
Alkahest with this latest version.

No, we don't get any kickbacks from Red
Hat, although their world headquarters are across the street from
ours. In the long run, we would to release AlkahEST for many
different platforms. We are just beginning to test it under Solaris
UNIX v9, GenToo Linux, and Max OS/X.

If you are working with Alkahest for
the first time, Red Hat 8.0 is by far your safest bet. Most of our
documentation - particularly the installation walkthrough - assumes
this platform. If you are willing to grit your teeth and try running
Alkahest on another platform, we'd love to get your feedback as we
strive to make Alkahest more widely available. However, you'll be
better equipped for the adventure if you successfully install and run
the system first, so in any case we recommend starting with Red Hat
8.0.

general
recommendations

We have several general recommendations
regarding installation:

- we recommend that you read through
  these instructions carefully before beginning the installation
  process.

- we recommend that, until such time
  as you are satisfied with the stability of the software, that you
  install it on a machine you don't need for anything else, and which
  doesn't contain sole copies of important files. This is prototype
  software, programmed by complete strangers who positively refuse to
  assure you that it has been sufficiently tested for production use.

- we recommend that you get help
  from someone with UNIX/Linux expertise. In this document we try to
  beat a path for you, but if you manage to find you've left the path
  you may need an experienced scout.

- we recommend that you post your
  problems to the Technical Support forum at
  http://www.alkahest.org/forums/
  , so that we (or another user) can try to help you, and so that we
  can work to remedy the problem for future releases.

Also, please give us as much feedback
as possible about how installation went (or went awry) for you. Any
feedback we get helps us to make the installation process easier in
future releases.

security
issues raised by installing Alkahest

Um, I know this is awkward, but before
we do this maybe we should have a little talk about *protection*.

If your machine is attached to the
network, and its web server is turned on, you have gone public. The
world is watching, or anyway it can be watching. It can also be
punching through your system's security vulnerabilities, spying on
you, or ravaging your data. Unless you want to put your career in the
hands of random antisocial

creeps, you take precautions.
Especially when you are running prototype web applications, like this
release

of Alkahest, which has not been closely
examined for security holes.

But even if we
could assume there are no security vulnerabilities *per se* in
Alkahest, you still don't want to

expose its
interface to the whole world. Anyone who can get their fingers on
those controls can irreversibly

command the system
to perform actions. Serving Alkahest's interface up to the web at
large means any random crazy person can delete your data sets.

If you are going to expose your
NuclearBLAST server to the World Wide Web, you will want to set up
Apache to serve NuclearBLAST up through an authenticated "Virtual
Server". This will mean that only registered users supplying
correct userid/password combinations will be able to access the web
application at all. You don't want mischievous unknown agents queuing
up BLAST searches on your server. Plus, there may be security holes
in this version: if there are, mischievous unknown agents might do
worse than set up BLAST searches. For testing, you have the option of
disconnecting your NuclearBLAST server from the network, because the
Apache web server should be able to send content to that machine's
own browser in the absence of a network connection. If you do this,
use 'localhost' as your hostname (and insert them wherever you see
hostnames referenced in the documentation below).

---

prerequisite
software

Alkahest relies on a lot of external software components to do its
work. All of the crucial components are freely available to academic
researchers. However, some of them cannot be bundled with our
Alkahest distributions, for licensing reasons. This helps make
installation a non-trivial process.

In most cases, we can just point you to the WWW or FTP sites that
hold the packages you need; in a couple of cases you may have to fill
out a form or email the authors of the software to get your hands on
it. Relax. Think how happy you'll be knowing that your data is being
processed by some of the very best academic software, and that it is
damn well on there too, because *you* installed it. That can be
an important emotional crutch when you find a piece that doesn't work
right away: whatever its problem is, it isn't that you failed to
install it, is what you can say.

Some of the components are only used in certain select areas of
the Alkahest system. If they are missing only a part of the system
will misbehave (i.e. fail, hang, crash, return an error message,
present you with strings in no human language, etc). Other components
are used ubiquitously, and if they are missing the system as a whole
cannot function. Take the following four components for example:

| Component type | What it does |
| --- | --- |
| MySQL-MAX Relational Database Management System (RDMS) v3.23.52 or greater | Organizes your data so that complex queries can be executed efficiently. |
| Apache HTT Server v2.0 or greater | The Web Server. Makes your data available to web browsers across a TCP/IP network. |
| PHP Hypertext processor v4.2 or greater | Web scripting language that underlies Alkahest's dynamic web pages. |
| Perl5 | Server-side language most Alkahest system internals are written in. |

If MySQL is missing a single-node Alkahest system will be a
mindless mess of scripts and web pages. If Apache is missing Alkahest
will mostly work, but will not be able to communicate anything to
users trying to reach it with their browsers. If the PHP processor is
missing, instead of useful web pages. the users' browsers will
display Alkahest's PHP program code served up as text files. If the
Perl interpreter is missing, many parts of the web interface may
appear to work fine, but Alkahest's system internals will be at a
halt. Basically everything else that has to be installed will only
cause limited malfunctions if it is missing or mis-installed. These
four'll kill ya.

What we've been liking about Red Hat
8.0 is that each of these four components may be easily installed
using Red Hat's distribution CDs. Get out your decoder rings and
prepare to receive special secret Red Hat installation instructions
shortly.

---

Red
Hat v8.0 installation

How easy do you want it?

There is an extremely easy way to
install Red Hat 8 that gives you most of the extras that Alkahest
needs. The problem is it gives you just about every other extra too,
and this bloats a system. It's not a bad idea to keep your production
Alkahest servers lean.

Below we supply you with a brief
walkthrough of a leaner installation we have used successfully. But
consider taking the ridiculously easy way if you are just interested
in getting Alkahest up and running so that you can take it for a spin
and see what you think of it. You can always do a more carefull
subsequent install later.

Installing Red Hat 8.0 the easy way:

During your Red Hat
Linux 8.0 install, select the "Custom System" option and
choose the package "Install Everything". This will probably
take up about 4.5 GB in your root-level (/) directory, so be mindful
of your storage limits.

Leaner meaner installation:

As above, choose the
"Custom System" option, but instead of installing
everything install whatever you think you'll need, making sure to
include all of the following packages (along with all the individual
packages listed under "Details"):

-- X-Windows

-- KDE and/or GNOME

-- Administration
Tools

-- System Tools

-- Web Server

-- SQL Database
Server

-- CPAN client (under
Development Tools)

After choosing these
packages, click the option "Choose individual packages". On
the next screen you should be able to find an RPM starting with
"php-mysql". Check it to include it, and then continue
through the installation process.

INSTALLATION LOCATION

To pave the way for installation that
is as painless as possible in this early release, we have assumed
that the package will be installed at the root level of your
filesystem (i.e. in the / directory). The package can be installed
elsewhere, but it will be easiest to follow the rest of these
instructions if you can abide by that location.

## NOTE: to install the package
elsewhere, you will have to replace /nuclearblast

## with the new location in files
/nuclearblast/etc/alkahest.xml and

## /nuclearblast/etc/my.cnf (which is
moved to /etc in the course of installation,

## see below.

GENERAL INSTALLATION OVERVIEW

AlkahEST NuclearBLAST requires the
interoperation of the following external applications and tools:

1. PHP (PHP Hypertext Processor)

2. Apache Web Server

3. MySQL-MAX v.23.49a-max+

4. BioPerl (and its CPAN dependencies)

5. NCBI BLAST

It's almost certain that some goofy
thing will differ between your system and ours, but we have done our
best to outline the configuration hoops we have run into. If you run
into any problems not addressed here, contact us through the
Technical Support forum at www.alkahest.org.

Alkahest Installation Walkthrough

These instructions assume that you are
logged into Red Hat 8.0 as the root user, and that the Alkahest
distribution tarball is in your system's root (/) directory.

1. Unzip the AlkahEST package

% tar -xzf nuclearblast-0.70.tar.gz

This should create a local directory
structure with a "root" level of /nuclearblast. Later on we
will store this location in an environment variable, $ALKAHEST\_ROOT
(see below)

2) Change permissions and ownership of
NuclearBLAST component directories (MySQL needs access to
/nuclearblast/innodb, and the Apache server needs access to
/nuclearblast/www.

% cd /nuclearblast

% chmod -R 755 .

% chown -R
mysql.mysql innodb

% chown -R
apache.apache www

% chown -R
apache.apache blastdbs

3. Configure MySQL

a) Replace the default my.cnf file with
our own version, and make any necessary adjustments to it.

% mv /etc/my.cnf /etc/my.cnf.old

% cp /nuclearblast/etc/my.cnf /etc

You should inspect the contents of this
file (/etc/my.cnf) using a text editor.

Our version may look similar to the
default that came with your system, but if you are already using
MySQL for other purposes you will want to choose your steps
carefully. MySQL documentation is avaiable at the MySQL web site
(http://www.mysql.com). The installation step outlined here is
appropriate if you are installing on a virgin Red Hat 8 system, as
recommended.

You may need to adjust these lines to
reflect your available RAM:

set-variable =
innodb\_buffer\_pool\_size=100M

set-variable =
innodb\_additional\_mem\_pool\_size=20M

If you have less than 256M, you may
want to set these lower; and any extra RAM you can allocate can only
help database performance. The rule of thumb we have been using in
development is to allocate 1/2 of the system's RAM to the database
here, divided into approximately the 5:1 proportions above. Keep in
mind that in a single-machine installation there is an inescapable
tradeoff between the RAM available to the database and the RAM
available to the computationally demanding BLAST processes. On many
systems it will probably be best to allocate a slimmer share of RAM
to the database, leaving more available for searches.

You will need to restart mysqld, the
mysql daemon, for any changes (including our replacement) to take
effect. Under Red Hat 8, you can do this using a desktop tool (follow
the menus: RedHat->Server Settings->Services). Check "mysqld"
if it is not checked already, highlight the mysql service and click
START at the top of the Service Configuration panel. If "mysqld"
IS checked already, click RESTART. From here it is also possible to
save a service configuration, so that you don't have to manually
restart all these services every time you reboot.

# NOTE: If you want to, you can launch
the daemon using the safe\_mysqld script

# that accompanies MySQL-MAX, which you
typically want to run in the

# background:

#

# % safe\_mysqld &

#

# Should you need to stop mysqld
manually:

#

# $ mysqladmin shutdown

#

# Lots of other fun command line
operations are explained in the

# MySQL documentation.

The first time you start the daemon, it
may take a minute or two for it (the daemon) to fully load, because
it is creating new files, big ones, in /nuclearblast/innodb/ibdata
and in /nuclearblast/innodb/iblogs. (By creating these big files
MySQL goes ahead and tries to reserve the disk space allocated to
InnoDB in /etc/my.cnf)

If there is something badly wrong with
the my.cnf file (for example if directories referenced do not exist,
or you don't have enough RAM to cover the stated allocations, etc),
then you will shortly get a message that mysqld has ended, meaning it
is NOT running, but has died. MySQL's error log file can then be
consulted, and usually it gives you a good indication of what the
problem might be:

% tail -f
/var/log/mysqld.log

If fortune shines on you, and the
daemon excecutes without any complaint, you have a running MySQL
database, blank except for the tables MySQL uses for its own
operations, and whatever test data set that may have accompanied the
distribution. You need to format yourself a NuclearBLAST database.

# NOTE: a NuclearBLAST database makes
use of MySQL's InnoDB tables, and with

# the my.cnf configuration file we have
given you, those tables are located

# under /nuclearblast/innodb (the files
MySQL reserves space with when you

# start the daemon.

Included in this distribution is a SQL
(Structured Query Language) file that contains all the instructions
MySQL needs to format a NuclearBLAST database. Formatting occurs
automatically when this file is "piped" to the MySQL
client:

% mysql <
/nuclearblast/sql/nuclearblast.sql

If this executes without a hiccup you
have created an (empty) NuclearBLAST database.

You now need to create a database user
(with a userid and passowrd) that has permission to read and write to
the database, which is simply called 'nuclearblast'. So invent
yourself a good userid and password, and get yourself to the MySQL
command prompt:

% bin/mysql

<mysql> use
mysql;

<mysql> GRANT
ALL ON nuclearblast.\* TO "userid@hostname" IDENTIFIED BY
"password";

(where your own
values replace -userid-, -hostname-, and -password-);

Now just remember the userid and
password, so that you can give this user's credentials to
NuclearBLAST (so it can manipulate the database). We'll cover that in
Configuring NuclearBLAST, below.

Installing
Bioperl

BioPerl is *the* repository of
Perl code for and by bioinformatics hackers. In future releases we
will surely owe an even greater debt to BioPerl, which we already use
to parse several file formats.

Obtain Bioperl v.1.0.2+ from
http://www.bioperl.org, and
follow the installation instructions at
http://www.bioperl.org/Core/Latest/index.shtml

Configuring
NCBI BLAST

We have included a recent distribution
of NCBI's BLAST software package. However, you will need to do one
thing to get it working. Copy the file .ncbirc from /nuclearblast/etc
to your system's root (/) directory:

% cp /nuclearblast/bin/.ncbirc /

Setting
environment variables

Or rather, variable. This package only
requires that you set one environmental variable: $ALKAHEST\_ROOT.
This variable holds the filesystem path to where the Alkahest package
is installed on your system. As mentioned earlier, these
installation instructions assume a root-level /alkahest directory.
In fact the package could be installed just about anywhere on the
system. For convenience the package relies on the $ALKAHEST\_ROOT
environment variable to know its own location in the file system.
We need that variable to be persistent -- we don't want to have to
set the variable every time we reboot -- and we might as well make it
sytem-wide, applying to all users (and any executables they own).

Setting
up a persistent system-wide shell environment variable

Under Red Hat 8.0 you may set a
persistent system-wide environmental variable by editing the
/etc/profile file. In this file you should a statement beginning with
'export', something like:

export USER, LOGNAME,
MAIL, HOSTNAME, HISTSIZE

Insert the following line immediately
after it, to specify that the $ALKAHEST\_ROOT environmental variable
for all users will refer to your /nuclearblast directory:

export
ALKAHEST\_ROOT=/alkahest

Restart the computer (or at least your
login shell) for these changes to take effect. Under BASH (Red Hat
Linux's default shell) you can verify whether they have by typing:

% echo $ALKAHEST\_ROOT

Setting
up the environmental variable for the Apache Web Server

Open the Apache configuration file
(/etc/httpd/conf/httpd.conf) in a text editor. Find the line that
defines the "ServerRoot". On a new line immediately
following this definition insert:

SetEnv ALKAHEST\_ROOT /nuclearblast

Restart httpd for this change to take
effect.

Preparing
Alkahest to be served

When your Apache web server is running,
everything stored “under” your /var/www/html directory will be
served up to the WWW (or whatever TCP/IP network your server is
connected to). We don't want to put the whole system there: that
would be quite insane. But we need to make sure Alkahest's web
content (HTML and PHP files) is in a directory that will be
Web-Served. You can configure Apache to serve up different
directories with different security restrictions, and if you are
going to use Alkahest in any sort of production capacity, you will
want to pay more attention to security details. For this
installation we are keeping things as simple as possible. We are
going to create a symbolic link to Alkahest's WWW document subtree
($ALKAHEST\_ROOT/www) in the existing Apache service directory
(/var/www/html):

% ln -s
/nuclearblast/www/nuclearblast /var/www/html

When you turn the web server on,
/var/www/html becomes the "Document Root" of the part of
the filesystem the server makes avaiable over the internet. Applying
this symlink effectively "points" the URL
http://your.domain.name.here/nuclearblast to the home directory of
NuclearBLAST's web components.

Starting
Apache

Now you just need to turn the web
server on if it isn't on already. Under Red Hat 8.0 the web server
daemon, httpd, may be started, stopped, and restarted using the same
GUI utility we used earlier to restart the mysqld daemon (menu:
RedHat->Server Settings->Services, choose httpd instead).
However, in

our experience if we do this Apache
isn't given the environmental variable we placed in the httpd.conf
file. We haven't figured out what causes this problem. The
workaround is to start, stop, and restart the daemon on the command
line, to wit:

% /sbin/service httpd
[start|stop|restart]

Configuring
*cron*

NuclearBLAST uses the CRON facility to
trigger the execution of any queued BLAST searches when resources
become available. To configure CRON to do this, you must add the
following lines to /etc/crontab:

ALKAHEST\_ROOT=/nuclearblast

0-59 \* \* \* \* root
/nuclearblast/bin/nb\_local\_Daemon.pl <hostname> <databasename>

In fact, a user with permission can
explicitly command the execution of any queued BLASTs with the
command:

%
/nuclearblast/bin/nb\_blastPIPE.pl <hostname> <databasename>

(Where <hostname> is replaced by
your system's hostname, and <databasename>, for the purpose of
this installation, is "nuclearblast".

Note however that you cannot perform
any BLAST searches until you create NuclearBLAST datasets by
importing sequence data in FASTA format (see Creating
New BLAST Datasets -- Alkahest User's Guide).

However, relying on the daemon is a
better idea, because it creates a pid file
(/var/tmp/NuclearBLAST.pid) that prevents another instance of
nb\_blastPIPE from launching a BLAST until the first instance is
finished. This should prevent your machine from grinding to a halt
under the demands of multiple simultaneous BLAST searches.

So that the changes you have made to
cron take effect, restart the crond daemon:

% /etc/rc.d/init.d/crond restart

troubleshooting
installation problems

TROUBLESHOOTING PHP

If you follow our recommended method of
installing Red Hat 8, PHP should automatically be seamlessly
integrated with Apache, and should be compiled with all the modules
NuclearBLAST needs. With any luck you won't have to do anything to
get PHP working. What follows in this section is a few notes for
people it doesn't seem to be working for!

Some users may find that they can get
the web server running, but that the PHP code isn't interepreted, but
is instead served up as text. That's a clue that PHP and Apache are
not working together. With one existing installation we (the
developers) have to explicitly request PHP in httpd's arguments when
we launch the daemon:

% /usr/sbin/httpd
-DHAVE\_PHP4

It is possible too that PHP modules
required by NuclearBLAST are not compiled into PHP on your system.
NuclearBLAST a couple of not-necessarily-standard PHP modules:

-- the mysql module,
which is used for accessing MySQL databases.

-- the GD module,
which is used for producing web graphics on-the-fly.

If your distribution of PHP is missing
these modules, you may want to try removing any pre-existing
installation of PHP, downloading the most recent stable release of
the source code from http://www.php.net, and compiling it with these
parameters (see PHP's documentation for more detailed instructions
for installing PHP):

% cd /usr/local/php
(or wherever you have unpacked the distribution)

% ./configure
--with\_mysql --with\_apxs --with\_gd

% ./make

% ./make install
