# Supplementary material for: Alkahest NuclearBLAST : a user-friendly BLAST management and analysis system
Source: BMC Bioinformatics. 2005 Jun 15;6:147. doi: 10.1186/1471-2105-6-147 (PMC1181624; doi:10.1186/1471-2105-6-147)
Supplement: Additional File 1 — The program, source and full documentation for installation are included. [file 1471-2105-6-147-s1.gz › alkahest-0.7.5/www/help/administrators_guide.html]

# Alkahest v0.7.4 Administator's Guide

Alkahest
server configuration

alkahest.xml
block/tag reference

Database
Definition blocks

Interceptor
Defaults block

Interceptor
Project Definition blocks

NuclearBLAST
configuration block

Checking Alkahest

Alkahest database backups

Alkahest
server configuration

alkahest.xml
tag reference

Database
Definition blocks

You can configure an Alkahest Web
Server's configuration file (alkahest.xml) to access any number of
Alkahest Data Servers. For each Alkahest Data Server you want to
access you must define a <database></database> block
within alkahest.xml. All tags described below are required.

| <hostname> | A valid hostname, i.e.  myhost.mydomain.com,  192.168.254.101,  localhost.localdomain, etc. | the hostname of the database |
| --- | --- | --- |
| <dbtype> | mysql | implementation type of RDBMS (presently "mysql" is only supported option) |
| <dbname> | Name given to an Alkahest database, for example just "alkahest" (sans quotes) | user-defined name of the database |
| <port> | 3306 | port used to communicate with the database (MySQL's port defaults to 3306). |
| <dbuser> | A mysql username (does not  have to be a user on the system, but must have been GRANTed access to <dbname> | a username with access to the database |
| <dbpasswd> | mySQL password associated with <dbuser> | the password dbuser uses to access the database |
| <sequencing> | 0 or 1 | Indicates whether database supports Interceptor |
| <blasting> | 0 or 1 | Indicates whether database supports NuclearBLAST |
| <annotations> | 0 or 1 | Indicates whether database supports Whiteboard (not yet implemented) |

Interceptor
Defaults block

| <mailbox\_dirs dir="dir1 dir2"> |  | Mailbox directories are directories you want Interceptor to intercept new volumes (directories) of trace files from. Interceptor must  have read/write access to these directories, and it will remove the volumes of trace files it finds there after it has deposited copies in the project's permanent storage location.    e.g: <mailbox\_dirs dir="/home/mike/chromats /home/sara/chromats"/> |
| --- | --- | --- |
| <cross\_match\_executable> |  | filesystem location of cross\_match, which must be executable by Interceptor. |
| <phred\_executable> |  | filesystem location of PHRED, which must be executable by Interceptor. |

These are the only tags that absolutely
must be in the <interceptor\_defaults> block. However, default
values for tags from the Project block can be defined by assigning
that value within <interceptor\_defaults>. Default values will
be overridden by any definitions present in that project's definition
block.

Interceptor
Project Definition blocks

Every Interceptor Project -- must have
its own project definition block within your alkahest.xml
configuration file. Interceptor determines what to do with new data
based on the alkahest.xml configuration parameters associated with
the project. In fact, Interceptor needs this file to be properly
configured to even recognize a set of data as belonging to a known
project. It does this by analyzing the names of the incoming reads
against each project's naming pattern, as defined within that
project's <pattern> tag:

| <pattern> | Some valid Perl regexp pattern, e.g.    <pattern>  RAD[^\_]+\_([A-H])(\d{2})\_xsay(\d{3})([a-d])([f|r])(\d)\_(\d{3})\.ab1  </pattern> | A (Perl regular expression) pattern that matches the naming convention for this project, and which also encloses in parentheses the various bits of data that have to be parsed from the name. |
| --- | --- | --- |

If your filenames match a pattern,
Interceptor gets to work. It needs to get a number of distinct pieces
of information from each name: the plate number, well location, and
so forth. Along with a correlate set of <get\_> tags, the
placement of parentheses pairs in your <pattern> indicates
where these datums live in the names. For example, the pattern in our
table above would match a batch of files named like:

RADEXAMPL\_A01\_xsay003af1\_001.ab1

RADEXAMPL\_A01\_xsay003af1\_002.ab1

RADEXAMPL\_A03\_xsay003af1\_003.ab1

..

It will 'yank' values into some special
variables defined by Perl wherever pairs of parentheses appear in the
pattern. According to our pattern seven elements are parsed from the
name as shown here:

RADEXAMPL\_(A)(01)\_xsay(003)(a)(f)(1)\_(001).ab1

Now interceptor needs to be told which
pair of parentheses encloses which datum. There are seven datums
(data, whatever) that Interceptor expects to be in each name: the row
of the well, the column of the well, the plate number, the plate
offset, the sequencing direction, the version number, and the
capillary number. Each of these datums has a "get\_" tag,
and the value for each tag should be the number of the
parentheses-pair from the original naming pattern.

It's something that is easier to
explain by example. Here are the "get\_" tags that would
correctly parse the necessary information from the sample filename we
referred to immediately above:

<get\_row>1</get\_row>

<get\_col>2</get\_col>

<get\_plate\_number>3</get\_plate\_number>

<get\_offset>4<get\_offset>

<get\_direction>5<get\_direction>

<get\_version>6<get\_version>

<get\_capillary>7<get\_capillary>

| <get\_row> |  | <pattern> parentheses pair bracketing  well row information. |
| --- | --- | --- |
| <get\_col> |  | <pattern> parentheses pair bracketing  well column information. |
| <get\_plate\_number> |  | <pattern> parentheses pair bracketing  plate number. |
| <get\_offset> |  | <pattern> parentheses pair bracketing  offset into a 384-well library plate. |
| <get\_direction> |  | <pattern> parentheses pair bracketing the sequencing read direction. |
| <get\_version> |  | <pattern> parentheses pair bracketing the sequencing read version. |
| <get\_capillary> |  | <pattern> parentheses pair bracketing the  capillary number. |
| <database\_host> |  | The name of the host of the Alkahest database that you want data from this project to be put into. (Each such database must have its own definition block). |
| <database\_name> |  | The name of the Alkahest database that you want data from this project to be put into. (Each such database must have its own definition block). |
| <project\_id> |  | The name of this sequencing project: should be a fairly short unique identifier. |
| <project\_location> |  | The filesystem location reserved for data from this project. The location must exist and Interceptor must have write access to it. |
| <problem\_dir>/alkahest/data/problems</problem\_dir> |  | The Problem Directory is the directory where you want Interceptor to deposit incoming data it could not handle (data whose naming pattern could not be correlated with any Project blocks in alkahest.xml, for example). Interceptor must have read/write access to this directory. |
| <log dir = "/alkahest/data/temp" /> |  | The Log Directory is the directory where Interceptor will deposit its .log files, names of which will look something like this:  INT-root-20030407-153902.log. Interceptor must have read/write access to this directory. |
| <admin\_email> |  | This tag encloses the email address of the Alkahest system administrator. |
| <alert\_email> |  | This tag encloses an email address to which alert messages are sent. |
| <alert\_treshold> |  | This tag should enclose a percent value (a value between 0 and 100). A plate whose percentage of "high quality" reads is less than this value triggers an alert email. |
| <phred\_value\_for\_HQ\_base> |  | This tag should enclose a PHRED-style quality value (a value between 0 and 100). This defines the minimum score necessary for a base to be considered "high quality". |
| <num\_allowable\_consecutive\_bad\_bases> |  | Establishes a number of bases not meeting the "high-quality" criteria, that will force the Trimmer to split the sequence by. Effectively defines the number of bad bases sufficient  to end a good read. |
| <primer\_file> |  | filesystem location of FASTA file containing primers to be screened in this project. Must be readable by Interceptor. |
| <vector\_file> |  | filesystem location of FASTA file containing vectors to be screened in this project. Must be readable by Interceptor. |
| <contaminant\_file> |  | filesystem location of FASTA file containing contaminants to be screened in this project. Must be readable by Interceptor. |
| <use\_contaminant\_screen> |  | yes/no. Indicates whether you want to screen the contaminants contained in <contaminant\_file> from your sequence. |
| <use\_polyA\_screen> |  | yes/no. Indicates whether you want Interceptor to screen for polyAs/polyTs. |
| <trim\_reads> |  | yes/no. Indicates whether you want Interceptor to trim the sequences. |
| <arrives\_in\_format> |  | Allowable values: 96-well, 384-well. Indicates what format you are expecting data to arrive in. For data straight off the ABI 3100 sequencers, this will be 96-well, i.e.:  <arrives\_in\_format>96-well</arrives\_in\_format> |
| <conversion> | Allowable values: from96to384, from384to384, from96to96, none. | Indicates what conversion should be performed on the read names. Generally 96-well names need to be converted back to the 384-well  format of an original library (a la from96to384). The other "from" options are supplied if you don't need to do that: "none"  is supplied if your names are already in exactly the format Interceptor will produce (probably only useful for running data through on subsequent occasions). |
| <project\_types> |  | This tag encloses a comma-separated list of strings, which are user-defined "types" that can be queried against, i.e.:    <project\_types>BAC, TEST\_DATA, PRIVATE</project\_types> |
| <project\_description> |  | This tag encloses a string that describes the project in basically any way you wish, as long as there aren't any weird XML-breaking characters in it. |
| <email\_list> |  | This tag encloses a comma-separated list of email addressed that are slated to recieve regular (non emegency-alert) emails about incoming data for this project. |
| <screen\_vector\_with\_crossmatch\_args> |  | A quoted argument string for crossmatch to use when screening vector. Ex:  <screen\_vector\_with\_crossmatch\_args>  "-minmatch 10 -minscore 20"  </screen\_vector\_with\_crossmatch\_args> |
| <screen\_contaminants\_with\_crossmatch\_args> |  | A quoted argument string for crossmatch to use when screening  contaminants. Ex:  <screen\_contaminants\_with\_crossmatch\_args>  "-minmatch 24 -minscore 20"  </screen\_contaminants\_with\_crossmatch\_args> |
| <dye\_chemistry> |  | The sequencing chemistry for this project. Presently only "big-dye" is supported (support consists of producing properly renamed St. Louis format trace files). Ex:  <dye\_chemistry>  big\_dye  </dye\_chemistry> |
