# Supplementary material for: Alkahest NuclearBLAST : a user-friendly BLAST management and analysis system
Source: BMC Bioinformatics. 2005 Jun 15;6:147. doi: 10.1186/1471-2105-6-147 (PMC1181624; doi:10.1186/1471-2105-6-147)
Supplement: Additional File 1 — The program, source and full documentation for installation are included. [file 1471-2105-6-147-s1.gz › alkahest-0.7.5/www/help/acknowledgements.html]

# Acknowledgements

We must acknowledge the efforts of all
who labored to produce the fine software we are using in our system
here. In an increasingly computer-mediated research community, truly
free and open science demands freely licensed and open source
software. Many research communities also have reason to seek out
cheap, or preferably *free* software. The free licensing
agreements of PHP, BioPerl, NCBI BLAST, and any other module or
application we incorporate is what makes this package possible.
Better, they make it possible to work for the day when the most
generally useful 'complete bioinformatics solutions' are the ones you
can download for free.

We'd also like to express our gratitude
to everone who has given us support, comments, code contributions,
testing, and general help; and especially to the persons listed
below:

NCSU
Center for Integrated Fungal Research (CIFR) / NCSU Fungal Genomics
Laboratory

Dr. Ralph Dean, Director

Dr. Tom Mitchell, Research Assistant
Professor

Doug Brown, Bioinformatics Lead

Dr. Michael Thon, Post-Doctoral
Scientist

Dr. Huaqin Pan, Bioinformatics
Researcher

NCSU
Center for the Biology of Nematode Parasitism

Dr. Charlie Opperman, Director

Mark Burke, IT/Bioinformatics Manager

Swedish
University of Agricultural Sciences Department of Forest Mycology and
Pathology

Dr. Fred Asiegbu, Professor

Lars Mattson

Mikael Lindberg

Jonas Hjelm

Dr. Yong-Hwan Lee, Director, Seoul
National University Fungal Plant Pathology Laboratory

Nigel Dunn-Coleman, Senior Researcher,
Genencor International, Inc.

Jason Stajich, Developer, BioPerl

Sheila Denn, Doctoral Student,
UNC-Chapel Hill School of Information and Library Science.
