# Supplementary material for: Alkahest NuclearBLAST : a user-friendly BLAST management and analysis system
Source: BMC Bioinformatics. 2005 Jun 15;6:147. doi: 10.1186/1471-2105-6-147 (PMC1181624; doi:10.1186/1471-2105-6-147)
Supplement: Additional File 1 — The program, source and full documentation for installation are included. [file 1471-2105-6-147-s1.gz › alkahest-0.7.5/www/help/users_guide.html]

# Alkahest v0.7.4 User's Guide

Interceptor

NuclearBLAST

Interpreting batch
NuclearBLAST results

Setting up batch
NuclearBLAST jobs

Creating
new NuclearBLAST datasets

Special instructions
for reconfiguring PHP to handle larger file uploads

How
to import a FASTA using the web interface

How
to import a FASTA on the command line

Special
instructions for importing NCBI release files as NuclearBLAST
datasets

Creating
new NuclearBLAST datasets

Alkahest NuclearBLAST does not come with all the BLAST datasets
you might want. However, you can make practically any FASTA-formatted
nucleotide or protein sequence file into an Alkahest NuclearBLAST
dataset. There are two principal methods for doing this. For small
files, you can use the web interface, simply uploading a FASTA file
and filling out a short form telling NuclearBLAST how it should be
formatted. Larger files should be inducted on the server's command
line.  
  
Why? Because file upload facilities can be exploited to
mount Denial Of Service (DOS) attacks on your web server, the PHP
engine on which Alkahest's web interface is built enforces limits on
the sizes of uploaded files. You can reconfigure PHP to raise this
limit (which probably has defaulted to somewhere between 2 and 8 Mb).
We can't generally recommend this procedure, but since it might make
sense for you to do this if you have taken extra care to limit
Alkahest web access to trusted users, we have outlined the procedure
below.  
  
For everyone else, adding a BLAST dataset on the
command line shouldn't be too much of a problem. We'll explain how to
do that too. We'll also tell you the best way to import some
widely-used target datasets available from NCBI.

---

### How to import a FASTA using the web interface

From the Alkahest Entry page you have to take a three-click trek
to the "importing a new set" page:

- > Click on the NuclearBLAST
  > database you want to add the dataset to.

- > Click **"manage
  > datasets"** in the menu at the top of the page.

- > Click on **"Import a new set"**.

You should arrive at a page titled **"Add a new BLAST
dataset"**, which contains a short form, through which
you must upload your FASTA file and tell NuclearBLAST a few things
about it:

- > First click the **<Browse>**
  > button to bring up a File Upload dialog (which allows you to select
  > a file on your local filesystem. Use it to find and select your
  > FASTA file. Once you do the pathname of that file should appear in
  > the text box next to the "Browse" button.

- > Click either the
  > "nucleotide" or "protein" radio button,
  > depending on the type of sequence data you are importing. Uploading
  > a nucleotide FASTA file and clicking "protein" will NOT
  > perform a translation for you; it will just screw you up.

- > As indicated by the
  > checkboxes on the form, by default your dataset will be available
  > both as a query and as a target. But you may want to restrict its
  > availability. For example, you might not want to allow a user to use
  > a very large dataset as a query (a self-BLAST of GenBank's NT
  > dataset is a very time-expensive proposition). So you can uncheck
  > either of these boxes if you'd like.

- > You're finished. Click the **<Submit File
  > for Validation>** button. It might take a few minutes to
  > induct your dataset, but within a few minutes it should be available
  > for BLAST searches. (i.e. it will appear as an available selection
  > for your Query or Target project when you use the web interface to
  > set up a batch BLAST job)

Again, unless you have re-configured PHP, this is only an option
for smaller FASTA files. If you attempt to upload a file that is too
large you will probably get an error page that says something like
this:

> Request entity too large!  
> The POST method does not
> allow the data transmitted, or the data volume exceeds the capacity
> limit.

In such a case you must either use the command-line method of
inducting BLAST datasets, or you must reconfigure the PHP
installation on your web server. Both actions require command-line
access to the web server; the latter one will probably require you to
have root (superuser) access.

---

### How to import a FASTA on the command line

The first step is to transfer your FASTA file to the filesystem of
your Alkahest server, using FTP, SSH, by running across the hall with
a floppy disk, etc. The second step is to execute the script
**nb\_add\_BLAST\_dataset.plx**. This script has seven
arguments, and they are all mandatory:

:   | **--dbhost** | hostname of the Alkahest database server |
    | **--dbname** | name of the Alkahest database |
    | **-i** | path/filename of your FASTA input file |
    | **-q** | (boolean) 1 if you want this dataset available as a query, 0 otherwise) |
    | **-r** | (boolean) 1 if you want this dataset available as a target, 0 otherwise) |
    | **-t** | [P/N] Sequence type: 'P' indicates Protein, 'N' indicates Nucleotide |
    | **-s** | A quoted string containing a short description of your data set |

> NOTE: $ALKAHEST\_ROOT is an **'environmental
> variable'** which should define the base location of your
> installation of Alkahest. It is possible that itis not defined for
> your user account, and it is possible that your user account is not
> authorized to execute the command. So if you can't seem to get this
> command to work, you will probably need to talk to your system
> administrator.

So for example, if you have a FASTA file of nucleotide sequences
called **myfile** in your **/tmp>**
directory, and you would like to make it available as both a query
and a target to a local Alkahest database named "alkahest",
your command might look like this:

> > $ALKAHEST\_ROOT/bin/nb\_add\_BLAST\_dataset.plx
> --dbhost localhost --dbname alkahest -i /tmp/myfile -q 1 -r 1 -t N -s
> "these are a few of my favorite DNA sequences"

If your FASTA file is very large, it may take some time for the
process to finish. (For example when I recently imported GenBank's NR
it took half an hour!) A lot of system and database activity will be
going on, and this may effect your system's performance temporarily.
For this reason you may elect to add large datasets at times when you
don't expect many users to be using Alkahest. There are special
considerations if you want to import FASTAs released by NCBI.

---

### Special instructions for handling NCBI datasets.

NCBI regularly releases updates of its vast archive on its FTP
site. Certain commonly used subsets of this archive, like "NR"
and "NT", are available as datasets formatted for use with
NCBI BLAST, as well as in plain FASTA files. It has been our
experience that the unformatted FASTA releases occassionally contain
header anomalies which confound NCBI's own BLAST dataset formatting
utility (formatdb).  
  
Because this is true, and because
Alkahest's own database needs information that it can only get from a
plain FASTA file, the safest way to induct NCBI's datasets into
Alkahest NuclearBLAST is slightly roundabout. First we'll give you a
summary of the procedure, and then we'll walk you through it.

### the simple summary

- > First, we download and
  > decompress a formatted BLAST dataset from NCBI.

- > We use NCBI's fastacmd
  > utility to "dump" the contents of that dataset to a FASTA
  > file.

- > We induct that FASTA into
  > the Alkahest system using the script nb\_add\_BLAST\_dataset.plx. This
  > script loads some critical data into the Alkahest database, and
  > creates formatted BLAST databases in the **<BLASTDB\_LOCATION>**
  > specified in the alkahest.xml configuration file.

- > We clean up by deleting all the files we downloaded,
  > decompressed, and dumped. All NuclearBLAST needs is the information
  > in its database and the corresponding formatted datasets that have
  > been inserted in the filesystem location specified by
  > **<BLASTDB\_LOCATION>**.

### walkthrough

**important preliminary note:** the files you will be
downloading are very large. As of the time of this writing GenBank's
uncompressed "NT" release takes up 9 Gigabytes of drive
space. Because we are going to be generating some very large working
files, we must work from a filesystem location that has significantly
more space than that! STEP ONE: downloading and decompressing
BLAST datasets from NCBI   
To get NCBI's datasets you use
NCBI's anonymous FTP facility. You can use a graphical client or a
web browser to do this, but since the rest of these instructions are
for the command line that's how we're going to tell you to FTP to
NCBI:

> `prompt> ftp ftp.ncbi.nlm.nih.gov`

You will be prompted for a userid; you just type "anonymous"
here. Then you will be prompted to supply your email address as a
password. After you do so, you should be left at another command
prompt, the FTP client's command prompt. Issue the following command
to move into the directory in which NCBI keeps its formatted BLAST
datasets:

> `ftp> cd /blast/db/FormattedDatabases`

Then download the database of your choice (brief descriptions of
your choices are available here).
If for example you were to download the "nr" dataset, you
would issue the command:

> `ftp> get nr.tar.gz`

Once the download has completed (it may take a while; again, these
files are very large!), just issue the command exit to exit
the ftp program.

> `prompt> fastacmd -D T -d /tmp/nr > /tmp/nr`

### 
