# Supplementary material for: Alkahest NuclearBLAST : a user-friendly BLAST management and analysis system
Source: BMC Bioinformatics. 2005 Jun 15;6:147. doi: 10.1186/1471-2105-6-147 (PMC1181624; doi:10.1186/1471-2105-6-147)
Supplement: Additional File 1 — The program, source and full documentation for installation are included. [file 1471-2105-6-147-s1.gz › alkahest-0.7.5/www/nuclearblast/help/nb_help_adding_BLAST_datasets_web.html]

Alkahest Help -- Adding a BLAST dataset using the web interface


### Adding a BLAST dataset using the web interface

From the Alkahest Entry page you have to take a three-click trek to
the "importing a new set" page:
> - Click on the NuclearBLAST database you want to add the dataset to.
> - Click **"manage datasets"** in the menu at the top of the page.
> - Click on **"Import a new set"**.

You should arrive at a page titled **"Add a new BLAST dataset"**, which contains a short form, through which you must upload your FASTA file and tell NuclearBLAST a few things about it:  
> - First click the **<Browse>** button to bring up a File Upload dialog (which allows
>   you to select a file on your local filesystem. Use it to find and select
>   your FASTA file. Once you do the pathname of that file should appear in the
>   text box next to the "Browse" button.
> - Click either the "nucleotide" or "protein" radio button, depending on
>   the type of sequence data you are importing. Uploading a nucleotide FASTA
>   file and clicking "protein" will NOT perform a translation for you; it will
>   just screw you up.
> - As indicated by the checkboxes on the form, by default your dataset
>   will be available both as a query and as a target. But you may want to
>   restrict its availability. For example, you might not want to allow a user
>   to use a very large dataset as a query (a self-BLAST of GenBank's NT
>   dataset is a very time-expensive proposition). So you can uncheck either
>   of these boxes if you'd like.
> - You're finished. Click the **<Submit File for Validation>** button. It might take a
>   few minutes to induct your dataset, but within a few minutes it should be
>   available for BLAST searches. (i.e. it will appear as an available selection
>   for your Query or Target project when you use the web interface to set up a
>   batch BLAST job)

Again, unless you have re-configured PHP, this is only an option for
smaller FASTA files. If you attempt to upload a file that is too large
you will probably get an error page that says something like this:
> Request entity too large!  
> The POST method does not allow the data transmitted, or
> the data volume exceeds the capacity limit.

In such a case you must either use the command-line method of inducting
BLAST datasets, or you must reconfigure the PHP installation on your web
server. Both actions require command-line access to the web server; the
latter one will probably require you to have root (superuser) access.
