# Supplementary material for: Alkahest NuclearBLAST : a user-friendly BLAST management and analysis system
Source: BMC Bioinformatics. 2005 Jun 15;6:147. doi: 10.1186/1471-2105-6-147 (PMC1181624; doi:10.1186/1471-2105-6-147)
Supplement: Additional File 1 — The program, source and full documentation for installation are included. [file 1471-2105-6-147-s1.gz › alkahest-0.7.5/www/nuclearblast/help/nb_help_adding_BLAST_datasets.html]

Alkahest Help -- Importing BLAST datasets


### Importing BLAST datasets to Alkahest NuclearBLAST

Alkahest NuclearBLAST does not come with all the BLAST datasets you might want. However, you can make practically any FASTA-formatted nucleotide or protein sequence file into an Alkahest NuclearBLAST dataset. There are two principal methods for doing this. For small files, you can use the web interface, simply uploading a FASTA file and filling out a short form telling NuclearBLAST how it should be formatted. Larger files should be inducted on the server's command line.  
  
Why? Because file upload facilities can be exploited to mount Denial Of
Service (DOS) attacks on your web server, the PHP engine on which Alkahest's
web interface is built enforces limits on the sizes of uploaded files. You
can reconfigure PHP to raise this limit (which probably has defaulted to
somewhere between 2 and 8 Mb). We can't generally recommend this procedure,
but since it might make sense for you to do this if you have taken extra care to limit Alkahest web access to trusted users, we have outlined the procedure below.  
  
For everyone else, adding a BLAST dataset on the command line shouldn't be
too much of a problem. We'll explain how to do that too. We'll also tell
you the best way to import some widely-used target datasets available from
NCBI.  
  
- How to import a FASTA using the web interface
- Special instructions for reconfiguring PHP to handle larger file uploads
- How to import a FASTA on the command line
- Special instructions for importing NCBI release files as NuclearBLAST datasets
