# Supplementary material for: Alkahest NuclearBLAST : a user-friendly BLAST management and analysis system
Source: BMC Bioinformatics. 2005 Jun 15;6:147. doi: 10.1186/1471-2105-6-147 (PMC1181624; doi:10.1186/1471-2105-6-147)
Supplement: Additional File 1 — The program, source and full documentation for installation are included. [file 1471-2105-6-147-s1.gz › alkahest-0.7.5/www/nuclearblast/help/nb_help.html]

Alkahest Help

Adding BLAST datasets on the command line

### Adding BLAST datasets on the command line

The first step is to transfer your FASTA file to the filesystem of your Alkahest server, using FTP, SSH, by running across the hall with a floppy disk, etc.
The second step is to execute the script **nb\_add\_BLAST\_dataset.plx**. This script has seven arguments, and they are all mandatory:
> |  |  |
> | --- | --- |
> | **--dbhost** | hostname of the Alkahest database server |
> | **--dbname** | name of the Alkahest database |
> | **-i** | path/filename of your FASTA input file |
> | **-q** | (boolean) 1 if you want this dataset available as a query, 0 otherwise) |
> | **-r** | (boolean) 1 if you want this dataset available as a target, 0 otherwise) |
> | **-t** | [P/N] Sequence type: 'P' indicates Protein, 'N' indicates Nucleotide |
> | **-s** | A quoted string containing a short description of your data set |
>
>   
> NOTE: $ALKAHEST\_ROOT is an **'environmental variable'** which should define the base location of your installation of Alkahest. It is possible that itis not defined for your user account, and it is possible that your user account is not authorized to execute the command. So if you can't seem to get this command to work, you will probably need to talk to your system administrator.

So for example, if you have a FASTA file of nucleotide sequences called **myfile** in your **/tmp>** directory, and you would like
to make it available as both a query and a target to a local Alkahest database named "alkahest", your command might look like this:
> > $ALKAHEST\_ROOT/bin/nb\_add\_BLAST\_dataset.plx --dbhost localhost --dbname alkahest -i /tmp/myfile -q 1 -r 1 -t N -s "these are a few of my favorite DNA sequences"

If your FASTA file is very large, it may take some time for the process to finish. (For example when I recently imported GenBank's NR it took half an hour!)
A lot of system and database activity will be going on, and this may effect
your system's performance temporarily. For this reason you may elect to add
large datasets at times when you don't expect many users to be using Alkahest.
There are special considerations if you want to import FASTAs released by NCBI.


Alkahest Help -- Importing BLAST datasets


### Importing BLAST datasets to Alkahest NuclearBLAST

Alkahest NuclearBLAST does not come with all the BLAST datasets you might want. However, you can make practically any FASTA-formatted nucleotide or protein sequence file into an Alkahest NuclearBLAST dataset. There are two principal methods for doing this. For small files, you can use the web interface, simply uploading a FASTA file and filling out a short form telling NuclearBLAST how it should be formatted. Larger files should be inducted on the server's command line.  
  
Why? Because file upload facilities can be exploited to mount Denial Of
Service (DOS) attacks on your web server, the PHP engine on which Alkahest's
web interface is built enforces limits on the sizes of uploaded files. You
can reconfigure PHP to raise this limit (which probably has defaulted to
somewhere between 2 and 8 Mb). We can't generally recommend this procedure,
but since it might make sense for you to do this if you have taken extra care to limit Alkahest web access to trusted users, we have outlined the procedure below.  
  
For everyone else, adding a BLAST dataset on the command line shouldn't be
too much of a problem. We'll explain how to do that too. We'll also tell
you the best way to import some widely-used target datasets available from
NCBI.  
  
- How to import a FASTA using the web interface
- Special instructions for reconfiguring PHP to handle larger file uploads
- How to import a FASTA on the command line
- Special instructions for importing NCBI release files as NuclearBLAST datasets


Alkahest Help -- Special instructions for handling NCBI datasets


### Special instructions for handling NCBI datasets.

NCBI regularly releases updates of its vast archive on its FTP site.
Certain commonly used subsets of this archive, like "NR" and "NT", are
available as datasets formatted for use with NCBI BLAST, as well as in
plain FASTA files. It has been our experience that the unformatted FASTA
releases occassionally contain header anomalies which confound NCBI's own
BLAST dataset formatting utility (formatdb).  
  
Because this is true, and because Alkahest's own database needs information
that it can only get from a plain FASTA file, the safest way to induct
NCBI's datasets into Alkahest NuclearBLAST is slightly roundabout. First we'll
give you a summary of the procedure, and then we'll walk you through it.

### the simple summary

> - First, we download and decompress a formatted BLAST dataset
>   from NCBI.
> - We use NCBI's fastacmd utility to "dump" the contents of
>   that dataset to a FASTA file.
> - We induct that FASTA into the Alkahest system using
>   the script nb\_add\_BLAST\_dataset.plx. This script loads some
>   critical data into the Alkahest database, and creates formatted
>   BLAST databases in the **<BLASTDB\_LOCATION>** specified in the
>   alkahest.xml configuration file.
> - We clean up by deleting all the files we downloaded,
>   decompressed, and dumped. All NuclearBLAST needs is the
>   information in its database and the corresponding formatted
>   datasets that have been inserted in the filesystem location
>   specified by **<BLASTDB\_LOCATION>**.

### walkthrough

**important preliminary note:** the files you will be
downloading are very large. As of the time of this writing GenBank's
uncompressed "NT" release takes up 9 Gigabytes of drive space.
Because we are going to be generating some very large working files,
we must work from a filesystem location that has significantly more
space than that!
STEP ONE: downloading and decompressing BLAST datasets from NCBI
  
To get NCBI's datasets you use NCBI's anonymous FTP facility. You can
use a graphical client or a web browser to do this, but since the rest
of these instructions are for the command line that's how we're going
to tell you to FTP to NCBI:
> `prompt> ftp ftp.ncbi.nlm.nih.gov`

You will be prompted for a userid; you just type "anonymous" here. Then you will be prompted to supply your email address as a password. After you do so,
you should be left at another command prompt, the FTP client's command prompt.
Issue the following command to move into the directory in which NCBI keeps its
formatted BLAST datasets:
> `ftp> cd /blast/db/FormattedDatabases`

Then download the database of your choice (brief descriptions of your choices are available here). If for example you were to download the "nr" dataset, you would issue the command:
> `ftp> get nr.tar.gz`

Once the download has completed (it may take a while; again, these files are very large!), just issue the command **exit** to exit the ftp program.
> `prompt> fastacmd -D T -d /tmp/nr > /tmp/nr`


Alkahest Help -- Adding a BLAST dataset using the web interface


### Adding a BLAST dataset using the web interface

From the Alkahest Entry page you have to take a three-click trek to
the "importing a new set" page:
> - Click on the NuclearBLAST database you want to add the dataset to.
> - Click **"manage datasets"** in the menu at the top of the page.
> - Click on **"Import a new set"**.

You should arrive at a page titled **"Add a new BLAST dataset"**, which contains a short form, through which you must upload your FASTA file and tell NuclearBLAST a few things about it:  
> - First click the **<Browse>** button to bring up a File Upload dialog (which allows
>   you to select a file on your local filesystem. Use it to find and select
>   your FASTA file. Once you do the pathname of that file should appear in the
>   text box next to the "Browse" button.
> - Click either the "nucleotide" or "protein" radio button, depending on
>   the type of sequence data you are importing. Uploading a nucleotide FASTA
>   file and clicking "protein" will NOT perform a translation for you; it will
>   just screw you up.
> - As indicated by the checkboxes on the form, by default your dataset
>   will be available both as a query and as a target. But you may want to
>   restrict its availability. For example, you might not want to allow a user
>   to use a very large dataset as a query (a self-BLAST of GenBank's NT
>   dataset is a very time-expensive proposition). So you can uncheck either
>   of these boxes if you'd like.
> - You're finished. Click the **<Submit File for Validation>** button. It might take a
>   few minutes to induct your dataset, but within a few minutes it should be
>   available for BLAST searches. (i.e. it will appear as an available selection
>   for your Query or Target project when you use the web interface to set up a
>   batch BLAST job)

Again, unless you have re-configured PHP, this is only an option for
smaller FASTA files. If you attempt to upload a file that is too large
you will probably get an error page that says something like this:
> Request entity too large!  
> The POST method does not allow the data transmitted, or
> the data volume exceeds the capacity limit.

In such a case you must either use the command-line method of inducting
BLAST datasets, or you must reconfigure the PHP installation on your web
server. Both actions require command-line access to the web server; the
latter one will probably require you to have root (superuser) access.
