# Supplementary material for: Alkahest NuclearBLAST : a user-friendly BLAST management and analysis system
Source: BMC Bioinformatics. 2005 Jun 15;6:147. doi: 10.1186/1471-2105-6-147 (PMC1181624; doi:10.1186/1471-2105-6-147)
Supplement: Additional File 1 — The program, source and full documentation for installation are included. [file 1471-2105-6-147-s1.gz › alkahest-0.7.5/www/nuclearblast/help/nb_help_adding_BLAST_datasets_cmd.html]

Alkahest Help -- Importing BLAST datasets on the command line


### Adding BLAST datasets on the command line

The first step is to transfer your FASTA file to the filesystem of your Alkahest server, using FTP, SSH, by running across the hall with a floppy disk, etc.
The second step is to execute the script **nb\_add\_BLAST\_dataset.plx**. This script has seven arguments, and they are all mandatory:
> |  |  |
> | --- | --- |
> | **--dbhost** | hostname of the Alkahest database server |
> | **--dbname** | name of the Alkahest database |
> | **-i** | path/filename of your FASTA input file |
> | **-q** | (boolean) 1 if you want this dataset available as a query, 0 otherwise) |
> | **-r** | (boolean) 1 if you want this dataset available as a target, 0 otherwise) |
> | **-t** | [P/N] Sequence type: 'P' indicates Protein, 'N' indicates Nucleotide |
> | **-s** | A quoted string containing a short description of your data set |
>
>   
> NOTE: $ALKAHEST\_ROOT is an **'environmental variable'** which should define the base location of your installation of Alkahest. It is possible that itis not defined for your user account, and it is possible that your user account is not authorized to execute the command. So if you can't seem to get this command to work, you will probably need to talk to your system administrator.

So for example, if you have a FASTA file of nucleotide sequences called **myfile** in your **/tmp>** directory, and you would like
to make it available as both a query and a target to a local Alkahest database named "alkahest", your command might look like this:
> > $ALKAHEST\_ROOT/bin/nb\_add\_BLAST\_dataset.plx --dbhost localhost --dbname alkahest -i /tmp/myfile -q 1 -r 1 -t N -s "these are a few of my favorite DNA sequences"

If your FASTA file is very large, it may take some time for the process to finish. (For example when I recently imported GenBank's NR it took half an hour!)
A lot of system and database activity will be going on, and this may effect
your system's performance temporarily. For this reason you may elect to add
large datasets at times when you don't expect many users to be using Alkahest.
There are special considerations if you want to import FASTAs released by NCBI.
