# Supplementary material for: Alkahest NuclearBLAST : a user-friendly BLAST management and analysis system
Source: BMC Bioinformatics. 2005 Jun 15;6:147. doi: 10.1186/1471-2105-6-147 (PMC1181624; doi:10.1186/1471-2105-6-147)
Supplement: Additional File 1 — The program, source and full documentation for installation are included. [file 1471-2105-6-147-s1.gz › alkahest-0.7.5/www/nuclearblast/help/nb_help_adding_BLAST_datasets_ncbi.html]

Alkahest Help -- Special instructions for handling NCBI datasets


### Special instructions for handling NCBI datasets.

NCBI regularly releases updates of its vast archive on its FTP site.
Certain commonly used subsets of this archive, like "NR" and "NT", are
available as datasets formatted for use with NCBI BLAST, as well as in
plain FASTA files. It has been our experience that the unformatted FASTA
releases occassionally contain header anomalies which confound NCBI's own
BLAST dataset formatting utility (formatdb).  
  
Because this is true, and because Alkahest's own database needs information
that it can only get from a plain FASTA file, the safest way to induct
NCBI's datasets into Alkahest NuclearBLAST is slightly roundabout. First we'll
give you a summary of the procedure, and then we'll walk you through it.

### the simple summary

> - First, we download and decompress a formatted BLAST dataset
>   from NCBI.
> - We use NCBI's fastacmd utility to "dump" the contents of
>   that dataset to a FASTA file.
> - We induct that FASTA into the Alkahest system using
>   the script nb\_add\_BLAST\_dataset.plx. This script loads some
>   critical data into the Alkahest database, and creates formatted
>   BLAST databases in the **<BLASTDB\_LOCATION>** specified in the
>   alkahest.xml configuration file.
> - We clean up by deleting all the files we downloaded,
>   decompressed, and dumped. All NuclearBLAST needs is the
>   information in its database and the corresponding formatted
>   datasets that have been inserted in the filesystem location
>   specified by **<BLASTDB\_LOCATION>**.

### walkthrough

**important preliminary note:** the files you will be
downloading are very large. As of the time of this writing GenBank's
uncompressed "NT" release takes up 9 Gigabytes of drive space.
Because we are going to be generating some very large working files,
we must work from a filesystem location that has significantly more
space than that!
STEP ONE: downloading and decompressing BLAST datasets from NCBI
  
To get NCBI's datasets you use NCBI's anonymous FTP facility. You can
use a graphical client or a web browser to do this, but since the rest
of these instructions are for the command line that's how we're going
to tell you to FTP to NCBI:
> `prompt> ftp ftp.ncbi.nlm.nih.gov`

You will be prompted for a userid; you just type "anonymous" here. Then you will be prompted to supply your email address as a password. After you do so,
you should be left at another command prompt, the FTP client's command prompt.
Issue the following command to move into the directory in which NCBI keeps its
formatted BLAST datasets:
> `ftp> cd /blast/db/FormattedDatabases`

Then download the database of your choice (brief descriptions of your choices are available here). If for example you were to download the "nr" dataset, you would issue the command:
> `ftp> get nr.tar.gz`

Once the download has completed (it may take a while; again, these files are very large!), just issue the command **exit** to exit the ftp program.
> `prompt> fastacmd -D T -d /tmp/nr > /tmp/nr`
